# The Potential Application of Pickering Multiple Emulsions in Food

**DOI:** 10.3390/foods11111558

**Published:** 2022-05-25

**Authors:** Iveta Klojdová, Constantinos Stathopoulos

**Affiliations:** Faculty of Agrobiology, Food and Natural Resources, Czech University of Life Sciences Prague, 165 21 Prague, Czech Republic; stathopoulos@af.czu.cz

**Keywords:** Pickering multiple emulsions, Pickering particles, food-grade, Janus emulsion, Janus particles

## Abstract

Emulsions stabilized by adsorbed particles—Pickering particles (PPs) instead of surfactants and emulsifiers are called Pickering emulsions. Here, we review the possible uses of Pickering multiple emulsions (PMEs) in the food industry. Food-grade PMEs are very complex systems with high potential for application in food technology. They can be prepared by traditional two-step emulsification processes but also using complex techniques, e.g., microfluidic devices. Compared to those stabilized with an emulsifier, PMEs provide more benefits such as lower susceptibility to coalescence, possible encapsulation of functional compounds in PMEs or even PPs with controlled release, etc. Additionally, the PPs can be made from food-grade by-products. Naturally, w/o/w emulsions in the Pickering form can also provide benefits such as fat reduction by partial replacement of fat phase with internal water phase and encapsulation of sensitive compounds in the internal water phase. A possible advanced type of PMEs may be stabilized by Janus particles, which can change their physicochemical properties and control properties of the whole emulsion systems. These emulsions have big potential as biosensors. In this paper, recent advances in the application of PPs in food emulsions are highlighted with emphasis on the potential application in food-grade PMEs.

## 1. Introduction

Multiple emulsions (also called double or complex) are systems of “emulsions in emulsions”. Multiple emulsions appear more commonly in the form w/o/w (water-in-oil-in-water), while o/w/o (oil-in-water-in-oil) are rarer. Because of the potential for fat reduction (w/o/w-partial replacement of fat content by internal water phase) as well as encapsulation efficiency of sensitive compounds (antioxidants, vitamins, etc.) or even microorganisms (probiotics) [1], multiple emulsions have a high potential for application in many industrial sectors, e.g., medicine, pharmacy [2], cosmetics [3,4], and food industry [5]. The application and preparation of multiple emulsions in food systems by surfactants (non-Pickering form) have been reviewed quite intensely [6,7,8,9,10,11,12,13]. Authors reported that the main limitation of these systems is lower stability than for simple emulsion systems. However, to our knowledge, no review was focused on Pickering multiple emulsions (PMEs) with potential applications in food.

w/o/w emulsions are very complex and usually have low thermodynamic stability [14]. The good stability and controlled encapsulation efficiency are still the main challenges for the wider application of multiple emulsions in food systems—especially w/o/w multiple emulsions. To ensure the prolonged stability and desirable behavior (encapsulation efficiency and controlled release of sensitive compounds), food-grade emulsifiers and stabilizers are required [15,16,17,18]. Unfortunately, the emulsifiers relevant for multiple emulsions preparation are often synthetic, and their addition in food is limited. An emulsifier for these food emulsions application is PGPR (E 476—polyglycerol polyricinoleate). PGPR is a mixture of products formed by the esterification of polyglycerols with condensed castor oil fatty acids, which has EFSA (European Food Safety Authority) “food safe status”, but its 2019 levels of addition in food areas are strictly regulated [19]. Its addition at the allowed levels would not be sufficient for the stabilization of multiple food emulsions. Moreover, there is now a trend to eliminate artificial additives, not excluding synthetic emulsifiers, and focus on natural sources of food improving substances [20,21].

Pickering emulsions are emulsions of any type and complexity, stabilized by solid particles instead of surfactants and emulsifiers. They were first described in 1907 [22]. The stabilization by solid particles (often called Pickering particles—PPs) brings specific properties—e.g., higher resistance to coalescence [23]. Generally, the stabilization of emulsion systems by biopolymer-based particles has been very popular in recent years, leading authors to use the term “neo-Pickering era” [24]. Another positive aspect might be the utilization of by-products from the food industry as a primary material for the preparation of Pickering particles. Pickering emulsions have been used in chemical and cosmetic industries, where inorganic particles can be applied [25]. However, there is still a gap in the preparation and application of food-grade PMEs. These emulsions a have high potential to deal with the big challenge of enhancing the potential quality of food products. Therefore, we present here this review focused on the feasibility of preparation and use of PMEs in food systems with a potential application as functional food or as advanced control systems in food technology.

## 2. Pickering Emulsions

The basic difference between the Pickering and “normal” emulsions is in their stabilization agents. Pickering emulsions are stabilized by solid particles on the interface of two immiscible liquids, whereas “normal” emulsions are stabilized by emulsifiers. The final stability of Pickering emulsions is then dependent on the particles’ composition and properties (type, shape, size, etc.) [26,27]. Pickering stabilization usually means the formation of strong film by colloidal solid particles on the interface [23]. These emulsions have then high physical stability and may even be used as a novel composite edible coating film with antifungal features [28].

### 2.1. Food-Grade Pickering Particles (PPs)

PPs are the key components in the preparation of Pickering emulsions. Food-grade PPs are limited because many particles do not remain insoluble and intact in both phases (water and oil) during the emulsion-lifetime period [29]. Many food-grade PPs have been discovered, and new materials are examined. Some materials enable not only stabilization but can also decrease water–oil interfacial energy and enhancement of functionality, such as oxidation resistance [30]. The use of Pickering emulsions in food systems has been discussed very intensively, and PPs in simple food emulsion systems have been reviewed many times [31,32,33,34,35,36,37,38,39,40,41,42]. The basic requirement is the food-grade status of used PPs and good stability of the resulting products. Some examples of primary material used in the last 5 years for food-grade PPs preparation and stabilization are summarized in Table 1. As established in the recent years of increased interest in Pickering emulsions, the use of these materials for the preparation of food-grade PMEs is a big challenge. The emulsifying ability of materials can also be dependent on molecular weight [43].

#### Potential Use of By-Products for Preparation of PPs

The added value for the environment can be the use of by-products from the food industry for the preparation of PPs. Usually, by-products from the food industry are milled and treated hydrothermally and can then be further used or treated for the preparation of PPs. For stabilization of emulsions, promising waste materials are ground coffee waste because of its lignin content [75] and whey protein, which has hydrophilic properties [72]. In general, the utilization of by-products can become an effective method to overcome the problems with the disposal of by-products [76]. Particularly, when by-products from the food industry contain considerable quantities of valuable and bioactive functional compounds, they are useful for both utilization and nutritional purposes [77]. PPs can then be carriers of bioactive materials (e.g., solid colloidal lipid particles with antioxidants) [78]. Moreover, in the area of bioactive compounds recovery from food by-products, there is a growing trend to use new eco-friendly methods—e.g., membrane-based technologies, ultrasound-assisted extraction, microwave-assisted extraction, nanotechnology, pulsed electric field, etc. Because of an effort to obtain required components using sensitive techniques, pulsed electric field and microwave are techniques with a high potential for use in the preparation of food-grade PPs. A pulsed electric field enables controlled mechanic damage and provides an economical and sustainable extraction of bioactive proteins. Another effective technique is the microwave. Microwave energy heats water molecules by ionic conduction and dipole rotation principles, and chemical components are then pushed out of the biomass. Generally, the targeted use of by-products obtained by eco-friendly methods contributes to sustainability. These new techniques provide minimally processed products. [79]. The contents of potentially bioactive compounds in different food products and the use of by-products from the food industry have been extensively reviewed by Galali et al. [80].

### 2.2. w/o and o/w Pickering Emulsions

**Figure 1:**
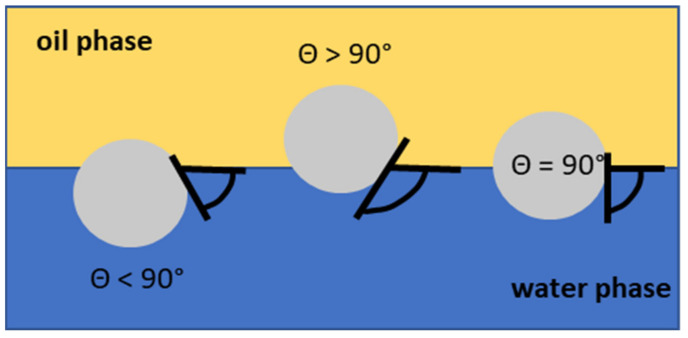
Examples of Pickering particles behavior on water–oil interface, determined by the wettability of solid particles.

In the emulsifier-stabilized emulsion type, the formed emulsion is basically defined by the hydrophilic–lipophilic balance value of the emulsifier [34,81,82]. The formulating Pickering emulsion type, w/o (water in oil) or o/w (oil in water), is determined by the wettability of solid particles (Figure 1). Particle wettability is defined by a three-phase contact angle (θ) [83]. o/w is created if a particle contact angle with the water phase is below 90° (hydrophilic particles), and w/o is created if a particle contact angle with the water phase is above 90° (hydrophobic particles). If a particle’s contact angle with the water and also oil phase is equal to 90°, the particle is anchored at the water–oil interface [27,35,84].

In general, the particles should have a defined degree of wettability, which ensures the appropriate interface absorption efficiency on the interface, and the particle size should be smaller than the droplet size of the prepared emulsion [85,86]. The wettability can be effectively tuned by physical and chemical surface modifications [87]. The typical range of PPs is in size from 5 nm to several μm [22]. The formation of PMEs can also be influenced by PPs shape. Due to the irregular surface of PPs (holes), nonspherical PMEs can then be formed [88,89].

## 3. w/o/w and o/w/o PMEs

**Figure 2:**
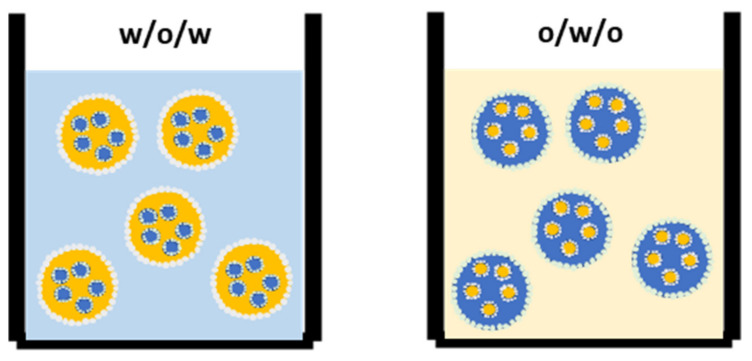
w/o/w and o/w/o PMEs with both interfaces stabilized by PPs (for simple and multiple emulsions).

**Figure 3:**
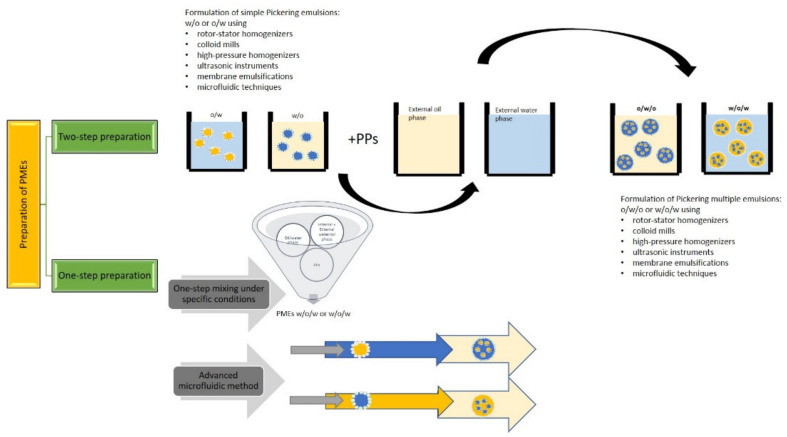
Schematic representation of possible PME preparations.

**Figure 4:**
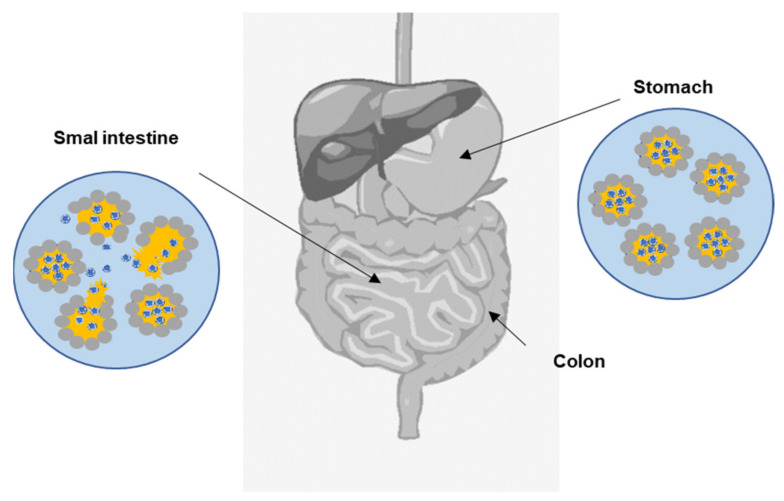
The scheme of the required behavior of w/o/w PMEs in gastrointestinal tract.

In the last decade, studies aimed at the preparation and stabilization of food-grade high-internal-phase simple Pickering emulsions. Some studies have been published, and this topic has been reviewed [27,90]. These emulsions have potential applications as fat substitutes and nutraceutical carriers [91]. However, they do not provide a complex structure for encapsulation. The advanced structures for the controlled delivery of sensitive compounds and fat replacement in food may be PMEs because of double interfaces (Figure 2). Those PMEs can be produced as shown in (Figure 3) and provide protection of an encapsulated internal phase in the stomach (Figure 4).

In general, w/o/w emulsions are the most used type of multiple emulsions. They offer the potential encapsulation efficiency levels and the protection of hydrophilic sensitive compounds in their internal water phase [7]. As such, the preparation of food-grade w/o/w PMEs has become the subject of interest. PPs can be used as either or as both of the internal or external water phase stabilizers. Often, PPs have been used as stabilizers for emulsions prepared with emulsifiers [92]. o/w/o emulsions are the less common type of multiple emulsions. They can be used mainly in cosmetics, especially for fragrance encapsulation [93]. Their wider applications in food may include encapsulation of essential oils and enhancement of fat content profile [12]. PPs could provide better stabilization of o/w/o PMEs because of the creation of a physical barrier that can inhibit fusion [94]. Here, the appropriate stabilization of the interfaces may be ensured by tiny fat crystals [95].

### 3.1. Stability of PMEs

Widely, the emulsion systems are thermodynamically unstable. Multiple emulsions, in particular, place higher demands on stabilization [7]. Naturally, the main barrier for PMEs is the presence of two interfaces. Here, we discuss the potential of food-grade PPs to ensure the sufficient stability of PMEs, which is necessary for their wider application in food technology.

#### 3.1.1. Stabilization of PMEs Only by PPs

If we focus on the PMEs prepared and stabilized only by PPs, the effectiveness of solid PPs depends on their wettability and morphology [34]. Other important parameters are the concentration of PPs, phase volume fractions of water and oil phases, the properties of water and oil phases, and order of addition during preparation processes [96]. In an ideal situation, in an emulsion effectively stabilized by PPs, their physicochemical properties must be very strictly defined, which is often unattainable for by-products from the food industry. The partially wetted particles in water and oil phases can adsorb on the interface and create a film, which can be single-layered or multi-layered. Some particles can also create 3D network [97,98]. These stabilizations usually prevent coalescence even when a creaming tendency is observed [99].

#### 3.1.2. Co-Stabilization of PMEs by Emulsifiers

Of course, it is also possible to partially replace PPs with synthetic emulsifiers. This option does not fulfill the total elimination of artificial additives but may be the first step towards improving the technology and making it more “green and nature friendly”. The most important aspect during the emulsion preparation, and especially mixing, is the order of addition of the various components and the avoidance of antagonism since PPs can be added to an emulsifier stabilized emulsion, or an emulsifier is added to a PP stabilized emulsion, or both can be added simultaneously. The order of addition of the PPs and emulsifiers enables the targeted preparation of emulsions due to controlled wetting of PPs [100]. The electrostatic interactions and also pH play a key role because of the influence on specific physico-chemical properties of emulsions (droplet size etc.). Therefore, especially for food-grade PMEs, the appropriate pH value of the whole system needs to be emphasized [101].

## 4. Preparation of Food-Grade PMEs

The most common preparation methods of PMEs are shown in Figure 3.

### 4.1. Two-Step Emulsification

To this date, two-step emulsification is still the most used technique for the preparation of multiple emulsions in general. In the traditional use of emulsifiers, internal (simple) w/o (hydrophobic emulsifier) or o/w (hydrophilic) emulsions are formulated (first step) and then consequently mixed in the second step with external water (hydrophilic emulsifier) or oil phase (hydrophobic emulsifier). For the two-step preparation of w/o/w PMEs, the procedure could be as follows: in the first step, the internal w/o emulsion is prepared by emulsifying water in the oil phase with dispersed hydrophobic PPs. In the second step, this emulsion is added to the external water phase with hydrophilic PPs, and both phases are mixed. Otherwise, the two-step preparation of o/w/o PMEs could be as follows: in the first step, the internal o/w emulsion is prepared by emulsifying oil in the water phase with dispersed hydrophilic PPs and then emulsified in the external oil phase with hydrophobic PPs [12,102].

For both steps of emulsification, rotor–stator homogenizers, colloid mills, high-pressure homogenizers, ultrasonic instruments, etc., are often used. These instruments use turbulence and/or cavitation processes and elongation to form emulsions. Another technique is a membrane emulsification method, which can replace these instruments or be incorporated into emulsification processes to improve particular properties of emulsions. Primarily, membrane emulsifications are less demanding on electricity consumption than the conventional emulsification instruments and permit the control of the size of the formed droplets. Usually, these methods use low pressure to force the dispersed phase to permeate through a membrane into the continuous phase [6,103,104]. An additional reduction of electricity consumption (almost 40% of energy) can be provided if an astatic mixer as a turbulence promoter is incorporated [105]. On the other hand, Scott et al., 2000 reported that a higher production velocity of emulsions (which is usually required) in cross-flow mode represents an increase in energy consumption costs [106]. The studies on membrane emulsification techniques are primarily focused on emulsions prepared using emulsifiers. However, the investigation of these methods for the application in the preparation of PMEs can be expected in the near future and could fulfill the requirements for the need to reduce energy consumption and utilization of natural products with the potential to stabilize PMEs. Several publications focused on the model application of emulsification membrane processes for Pickering emulsions preparation mentioned enhanced coalescence [107,108]. Huang et al., 2022 reported the preparation of Pickering emulsions using membrane emulsification at appropriate pH conditions [109]. One of the options for improving the stability of Pickering emulsions prepared by the membrane technique is a partial replacement of emulsifiers by PPs [110].

### 4.2. Advanced Methods

#### 4.2.1. One-Step Emulsification

PMEs can also be prepared in a single step. All three phases (for w/o/w PMEs: internal water phase, oil phase, external water phase) and the PPs are mixed concurrently, and the formation of PMEs is ensured by changes in physico-chemical properties of the whole system (pH, temperature, phase volumes) [111]. Usually, these preparations require specific properties of used phases. Generally, one-step emulsification of w/o/w PMEs can be realized using very high viscosity silicone oil (≥10,000 cSt) and modified silica particles [112]. Unfortunately, these materials are unsuitable for food products. One published promising combination of PPs for food-grade emulsions is a mix of Arabic gum with gliadin nanoparticles where a one-step emulsification process avoided aggregation of PPs, which can occur before emulsification. These systems may be an appropriate solution for the controlled formation of PMEs in one step. Arabic gum has amphiphilic properties and can stabilize both interphases [113]. An example of the one-step formation of food-grade w/o/w PMEs is the total replacement of synthetic emulsifiers by corn-peptide-functionalized particles with adaptable wettability, which can be controlled by the composition of the oil phase. The preparation was realized by mixing all phases and PPs, and the mixture was then mixed by a homogenizer only. This specific formulation of PMEs has a promising potential to be applied in the food industry. However, these preparations need a deeper study because of the specific composition of these systems [114].

#### 4.2.2. Microfluidic Methods

Microfluidic techniques for multiple emulsions preparation have become an object of interest in the last decade and have been reviewed recently [115,116]. These techniques allow “custom emulsification” (low polydispersity, 100% encapsulation efficiency, number of droplets, droplet sizes, etc.) [117,118]. A model preparation of food-grade w/o/w emulsions for the reduction of sugar content in food using the microfluidic technique was recently published [119]. Generally, preparations in microfluidic devices involve an injection of the dispersed phase through the microchannel into another microchannel containing the continuous phase [120]. Essentially, this technique can be used for either single or two-step emulsification processes. The advanced special microfluidic devices enable the preparation of multiple emulsions also in one step [116]. The experimental formation of o/w Pickering emulsions prepared using the microfluidic technique and stabilized by hydrophilic silica particles showed that this technique enables the preparation of stable systems [121] and could also be used for PMEs. The application of microfluidic techniques for the preparation of food-grade PMEs requires more detailed studies in this field. The main disadvantage of these techniques is the lower production rates due to their slow speed (for a single drop maker <1 mL/h) [115].

## 5. Potential Application of PMEs in Food Systems

### 5.1. Low-Fat Products

The food industry is now continuously developing new products that are focused on human health and diet. The most prevailing concern in developed countries is obesity and high fat intake [122]. Wang et al., 2018 used cellulose-nanofiber-based Pickering emulsion and were able to decrease the fat content in sausages [123], while Xie et al., 2021 [124] showed the potential of simple cellulose-based Pickering o/w emulsions to reduce fat content in biscuits.Moreover, an additional stabilizing effect and improvement of dietary properties were provided by bamboo shoot fiber [124]. The partial replacement of butter with cinnamon essential oil in zein stabilized Pickering emulsions, enhanced nutritional value, and facilitated better control of mold growth in food products [125]. Partial replacement of the oil phase by an internal water phase in food-grade Pickering multiple w/o/w emulsions permitted an additional reduction of fat content. Although the presence of two interfaces reduces the thermostability of the whole system [126], the content of PPs is higher and may raise dietary properties (e.g., higher content of fiber). Unfortunately, there are not any complex studies describing a model preparation of food-grade PMEs; however, the partial replacement of emulsifiers by quinoa starch particles during the preparation of multiple w/o/w emulsions has already been investigated [127]. One published study demonstrates the potential of model food-grade Pickering emulsions to significantly reduce the extent of lipolysis due to stabilization by ovotransferrin and lysozyme [56]. This stabilization could also provide a relevant extension of multiple emulsion oxidation stability.

### 5.2. Functional Food with Encapsulated Compounds

The preparation of emulsifier-free PMEs with encapsulated sensitive compounds and good stability is probably the biggest challenge in this research area. These emulsions could be considered drug delivery carriers without any negative side effects. Boostani et al., 2022 [128] published the preparation of w/o/w PMs with encapsulated vitamins in the internal water phase and stabilized by hordein nanoparticles. Jiang et al., 2021 [129] described food-grade w/o/w PMEs stabilized by zein particles and lecithin, which showed their synergistic effect.

Moreover, bioactive compounds can be involved in PPs, which stabilize interfaces of Pickering emulsions. The encapsulation of curcumin in cellulose nanocrystals, which stabilized Pickering o/w emulsions, was recently published [130]. Thus, PMEs offer the possibility of encapsulation of sensitive compounds in the internal phase (internal water phase for w/o/w and internal oil phase for o/w/o), and also, an additional benefit could be the incorporation of bioactive compounds in PPs. This possibility means an advantage of PMEs compared to conventional emulsions prepared with emulsifiers only.

### 5.3. Janus Particles and Emulsions

An advanced type of PPs are Janus particles, which enable controlled changes in the stability and type of Pickering emulsions [34]. Janus particles have at least two different chemical or physical properties on their surface, and their shape can be spherical, cylindrical, snowman-like, etc. [131]. The name Janus comes from Roman mythology, where Janus is a god with two faces [132]. The application of Janus particles in food systems has not been fully explored yet. However, promising materials for further surface modification to prepare Janus particles for emulsion systems could be starch and chitosan, which offer additional antimicrobial properties when used in combination with silver [133,134]. The preparation of highly stable model Janus multiple w/o/w emulsions by a one-step technique using stimuli-responsive amphiphilic Janus particles as emulsifiers was demonstrated. The authors presented the use of Janus particles as an advanced solid PPs-emulsifier with additional properties [135]. These Janus particles also provide controlled destabilization of emulsion systems in response to external stimuli. The triggered release of encapsulated compounds in internal water phases could be managed by pH changes. Unfortunately, for the application of these systems in food, a lot more detailed studies are needed in this area. Further microscale studies are proposed to describe the mechanism of the observed formation and stabilization [135].

Additionally, the use of Janus emulsions as biosensors for the detection of pathogens has been reported. They could be used as alternatives to commercial agglutination assays [136]. This research area opens new approaches for food quality monitoring. In the future, the use of Janus emulsion in food technology may also be a laboratory tool for pathogen identification. The use of multiple emulsion systems may be studied in the future because of the possible encapsulation of antimicrobial agents. These systems could be used for pathogen detection and targeted destruction. Therefore, the application of PMEs in the food industry is very promising because of their potentially wide application. However, these formations and uses of Janus multiple emulsions require deep studies.

### 5.4. In Vitro Behaviour of PMEs

For PMEs application in food systems, their behavior in the gastrointestinal tract plays a key role. The gastrointestinal tract is the system of organs that ingests, digests food, extracts energy and nutrients from it, and expels remaining waste [137]. Many models of in vitro gastrointestinal tract were studied. Some models are static [138,139], but dynamic models are more authentic [140,141]. Currently, there is a tendency to develop models based on computer-controlled simulations of the complex digestion processes [142]. Basically, the most important parts for the simulation are the stomach and intestine, and the detailed protocol of this simulation was described by Brodkorb et al., 2019 [143]. After consumption, the crucial digestion process for PMEs is in the stomach, where it is necessary to ensure the stability of primary emulsions with encapsulated sensitive compounds. Oral digestion usually has a minor impact on Pickering emulsions [144]. Because of low pH and pepsin in the stomach, PPs based on protein can be denatured and decrease the stability of the whole system. Usually, food stays in a peristaltically moving stomach between 0.5 and 4 h, and the pH is around 1–3. The most published change in food emulsions when passing through the stomach is protein hydrolysis [145,146]. In general, the intestinal part pH is between 6 and 7.5, and enzymes (lipase, trypsin, chymotrypsin), co-enzymes, inorganic salts, and bile salts with surface activity are presented. Often, under these conditions, emulsion droplets aggregate [147]. Bile salts can displace adsorbed proteins and facilitate lipase adsorption on oil droplet surfaces, which can result in significant coalescence [148] and disruption of emulsions. Generally, for multiple w/o/w emulsion systems, the release of the internal water phase and absorption of sensitive compounds in the intestine is required. Commonly used food-grade emulsifiers (e.g., PGPR) for w/o stabilization meet this requirement. Actually, there is a lack of studies focused on the stabilization of w/o emulsions by PPs and the description of their behavior in in vitro simulated gastrointestinal tract. However, stabilization of w/o emulsions by fat crystals and their in vitro behavior was published. Fat crystals are often considered as PPs [149]. The schema of the desirable behavior of w/o/w PMEs is shown in Figure 4. On the contrary, many studies on o/w were published [150,151,152,153]. Xiao et al., 2017 [154] reported the digestion behavior of w/o/w emulsions prepared using a PGPR emulsifier (internal w/o emulsions) and stabilized by PPs (outer w/o/w emulsions). Edible kafirin nanoparticles were used as PPs. During gastric fluid simulation, w/o/w emulsions underwent structural changes, flocculation was observed, and the internal water phase was released from some particles. The authors assumed the digestion of kafirin nanoparticles by protease. Then, in simulated intestinal fluids, a transition of w/o/w in w/o structure was observed. The preparation of food-grade PME with required behavior in the gastrointestinal tract remains a big challenge. Above that, the controlled release of bioactive compounds from PPs in the gastrointestinal tract could also be implemented. This could be used during colon cancer therapy [130].

The potential of functional food prepared with PMEs for application in medicine seems to be very promising. It is worth mentioning that Cai et al., 2020 reported the potential application of Pickering emulsions with antibacterial and anti-inflammatory effects [155].

## 6. Conclusions

This review summarizes the findings in the field of the potential application of PMEs in food. Generally, Pickering emulsions have been a hot topic in recent years and found their application in material engineering, medicine, cosmetics, and food technology. The main reason why they are so popular is the current trend to cut down the use of artificial additives in the food industry and focus on the utilization of by-products. By-products can thus be used efficiently, and food technology becomes more environmentally friendly. Moreover, functional products are very popular on the market, and PMEs offer additional effects (encapsulation of sensitive materials, lower fat content, etc.). To date, there are only a few studies focused on model preparation of food-grade PMEs. Thus, the first step to a wider application of food-grade PMEs may be the effort to partially replace synthetic emulsifiers with PPs. In summary, we feel that PMEs have a big potential to be widely used in the food industry. Additionally, advanced Janus formulations may become sensors for contamination (e.g., microbial) in food operations. However, future investigations on the preparation, stabilization, and properties of food-grade PMEs are needed. Future research attention may also be directed to Janus particles.

## Figures and Tables

**Table 1:** Examples of recently published studies on food-grade PPs for preparation and stabilization of simple emulsions.

PPs for Simple Emulsions—Primary Material	Reference
Apple pomace	[44]
Bamboo shoot	[45]
Casein	[46,47]
Cellulose	[48,49,50,51]
Chitosan	[52,53]
Egg proteins	[54,55,56,57]
Gelatin	[58,59]
Lupin cultivar	[60]
Pea protein	[61]
Peanut protein	[62]
Quinoa protein	[63]
Rapeseed protein	[64]
Starch	[65,66]
Soy protein	[67,68]
Tea components	[69,70]
Walnut flour	[71]
Whey protein	[72]
Zein	[73,74]

## Data Availability

Not applicable.
